# Triglyceride-Glucose Index as Predictor for Hypertension, CHD and STROKE Risk among Non-Diabetic Patients: A NHANES Cross-Sectional Study 2001–2020

**DOI:** 10.1007/s44197-024-00269-7

**Published:** 2024-07-02

**Authors:** Bisher Sawaf, Sarya Swed, Hidar Alibrahim, Haidara Bohsas, Tirth Dave, Mohamad Nour Nasif, Wael Hafez, Fatema Ali Asgar Tashrifwala, Yazan Khair Eldien Jabban, Safwan Al-Rassas, Heba haj Saleh, Abdul Rehman Zia Zaidi, Baraa Alghalyini, Shaymaa Abdelmaboud Mohamed, Waleed Farouk Mohamed, Amr Farwati, Mohammed Najdat Seijari, Naim Battikh, Basma Elnagar, Seema Iqbal, Karla Robles-Velasco, Ivan Cherrez-Ojeda

**Affiliations:** 1 Department of Internal Medicine, Hamad Medical Corporation, Doha, Qatar; 2 Faculty of Medicine, Aleppo University, Aleppo, Syria; 3 Bukovinian State Medical University, Chernivtsi, Ukraine; 4 NMC Royal Hospital, 16Th Street, Khalifa City, Abu Dhabi, UAE; 5 Department of Internal Medicine, Medical Research and Clinical Studies Institute, The National Research Centre, Cairo, Egypt; 6 Department of Research and Discovery, Stamford Health, Stamford, USA; 7 Faculty of Medicine, Damascus University, Damascus, Syria; 8 Faculty of Medicine, Thamar University, Dhamar, Yemen; 9 Department of Family and Community Medicine, College of Medicine, Alfaisal University, Riyadh, Saudi Arabia; 10 Department of Cardiology, National Heart Institute, Ibn Al Nafees Square, AL KIT KAT, Agouza, Giza Governorate Egypt; 11 Healthpoint Hospital, Abu Dhabi, UAE; 12 John H. Stroger, Jr. Hospital of Cook County, Chicago, USA; 13 Lecturer of Cardiovascular Medicine, Cardiovascular Department, Faculty of Medicine, Tanta University, Tanta, Egypt; 14 Khyber Medical College, University of Peshawar, Peshawar, 25120 Khyber Pakhtunkhwa Pakistan; 15 Universidad Espíritu Santo, Samborondón, Ecuador; 16 Respiralab Research Group, Guayaquil, Ecuador; 17 Independent Researcher, THE GLOBEST TEAM , https://www.theglobestteam.com/; 18 Department of Cardiology, Al Salam Specialist Hospital, Building 1, Road 39, Block 941, Riffa, 80278 Bahrain

**Keywords:** TyG index, Cardiovascular diseases, NHANES

## Abstract

**Background:**

Cardiovascular disease (CVD) is a leading cause of global mortality. Early intervention and prevention of CVD depend on accurately predicting the risk of CVD. This study aimed to investigate the association between the TyG index and the risk of coronary heart disease (CHD), congestive heart failure (CHF), heart attack (HA), stroke, and hypertension (HTN) among patients without diabetes in the United States.

**Methods:**

In this retrospective, cross-sectional study, we used data from the National Health and Nutrition Examination Survey (NHANES) from 2001 to 2020. We conducted several regression analysis models and calculated the sensitivity and specificity of (TyG) index for predicting the onset of CHD, CHF, HA, stroke, and HTN.

**Results:**

A total of 10,937 individuals without diabetes participated in our study. Individuals with a TyG index greater than 8.96 displayed significant increasing in various parameters, including BMI, systolic/diastolic blood pressure, total cholesterol, LDL, and Apo-B levels (*p* < 0.001). Almost all regression models ensured that a higher TyGI value was associated with higher odds of having CHD, CHF, HA, stroke, and HTN, which patients with a TyGI value higher than 8.96 have odds ratios of 2.24–5.58 for CHD, 1.68–4.42 for stroke, 2.45–3.77 for HA and 1.75–3.93 for HTN comparing than patients with a TyGI value lower than 8.11 (p-value < 0.05).We evaluated the predictive value of the TyG index for each endpoint, obtaining the following area under the curve (AUC) values: 54.75% for CHF (95% CI: 0.542–0.614), 52.32% for stroke (95% CI: 0.529–0.584), 55.67% for HA (95% CI: 0.595–0.646), 55.59% for HTN (95% CI: 0.574–0.597), and 50.31% for CHD (95% CI: 0.592–0.646).

**Conclusion:**

The TyG index showed a strong correlation with cardiovascular risk factors in individuals without diabetes, however it was a poor predictor of almost studied cardiovascular diseases.

## Background

Cardiovascular disease (CVD) consists various conditions affecting the heart and blood vessels, including coronary heart disease, cerebrovascular disease, rheumatic heart disorder, peripheral arterial disease, congenital heart disease, and deep vein thrombosis. CVD is the leading cause of mortality worldwide, particularly in developing countries [1]. In the United States, one person dies from CVD every 33 s, resulting in 695,000 deaths by 2021 [2]. Despite continuous global efforts to reduce CVD risk, its high burden remains because of complex risk factors that necessitate long-term lifestyle modifications and pharmaceutical interventions.

Determining the optimal standard for assessing the risk of CVD is challenging due to variability among risk groups, limited comparable categories, and diverse characteristics of individuals classified as 'at-risk' [3].

Several studies have demonstrated that insulin resistance (IR) plays a significant role in the development of cardiovascular disease (CVD) in both individuals with and without diabetes mellitus [4–6]. Therefore, insulin resistance (IR) has been recognized as a predictor of cardiovascular disease (CVD) in both healthy and populations with diabetes mellitus [7]. Additionally, IR can stimulate sympathetic nervous system activity and indirectly increase the efficacy of the renin–angiotensin–aldosterone system, leading to water-sodium retention and vascular hyperactivity [6]. The gold standard for diagnosing IR is the hyper-insulinemic-euglycemic clamp. However, due to the complex testing process and high cost, it is not commonly used in large-scale epidemiological studies.

In contrast, the triglyceride-glucose (TyG) index has been suggested as an alternative, low-cost, and time-saving tool, particularly in some clinical epidemiological studies [5]. According to previous studies, carotid atherosclerosis, coronary artery calcification, and high risk of CVD are correlated with the triglyceride glucose (TyG) index [8]. Many studies have proposed a correlation between the TyG index and the risk factors for CVD and all-cause mortality in patients with CVD [9].

Few studies have examined the relationship between the TyG index and hypertension [6]. Furthermore, no study has investigated whether the TyG index can predict the risk of hypertension in healthy individuals. In addition, there is an urgent need to predict the risks of CVD, HTN, and stroke in the early stages to minimize mortality, morbidity and financial burden. This American dataset cross-sectional study aimed to discover the clinical and statistical correlation between TyG index and the risk of coronary heart disease, congestive heart failure, stroke and hypertension in patients without diabetes mellitus.

## Methods and Materials

### Study Design and Population

This study analyzed data from the National Health and Nutrition Examination Survey (NHANES), a comprehensive survey conducted every two years to assess the health and nutritional status of adults and children in the United States through physical examinations, interviews, and laboratory tests. To account for non-response and sample design, the NHANES employs a complex multistage sampling design that requires the use of sample weights for accurate representation.

Data from 10 cycles (2001–2002, 2003–2004, 2005–2006, 2007–2008, 2009–2010, 2011–2012, 2013–2014, 2015–2016, 2017–2018, and 2019–2020) were merged, and the data analysis was performed using a 4-year fasting weight (WTSAF4YR) following the NHANES analysis criteria. The NHANES was conducted by the Centers for Disease Control and Prevention (CDC) and the National Center for Health Statistics (NCHS). The research ethics review committee of the NCHS approved the study. Written informed consent was obtained from all the respondents. The NHANES data utilized in this study were retrieved from DataDryad [10]. The study sample included participants without diabetes mellitus aged ≥ 18 years. Individuals with diabetes and missing data on the triglyceride-glucose index (TyG index) were excluded from the study. Incomplete data on hypertension, coronary heart disease (CHD), congestive heart failure (CHF), or stroke were set as missing to eliminate their effects on the analysis. The final study population comprised of 10,937 individuals. A flow chart detailing the specific study population is presented in “Additional File S1”.

## Data Collection and Definitions

Sociodemographic data (age, sex, race/ethnicity, education, marital status, alcohol consumption, working hours, vigorous activity, and smoking), physical examination (height, waist circumference (WC), weight, heart rate (HR), and body mass index (BMI)), medication history (antihypertensive drugs, cholesterol-lowering drugs, and daily low-dose aspirin), and laboratory test results (direct HDL-cholesterol, total cholesterol, LDL-cholesterol, apolipoprotein B, urinary albumin, and urinary creatinine) were collected through a questionnaire. BMI was calculated as the natural logarithm of [weight (kg)/height (m2)]. Blood pressure (BP) was measured by two doctors using a standard mercury sphygmomanometer, and a minimum of three measurements was averaged to determine the value. The triglyceride-glucose index (TyG index) was calculated as the natural logarithm of [fasting triglycerides (mg/dL) × fasting glucose (mg/dL))/2]. Hypertension was defined based on physician-diagnosed or self-reported use of antihypertensive medications. Congestive heart failure was defined based on self-reported physician diagnoses. Coronary heart disease (CHD) was defined based on a self-reported physician’s diagnosis of heart attack or myocardial infarction. Stroke was defined based on a self-reported physician’s diagnosis of stroke or transient ischemic attack. Heart attack was defined based on a presence of history of acute coronary syndrome, myocardial infarction or coronary artery disease. The endpoints of this study were the incidences of CHD, CHF, stroke, HA, and hypertension. These endpoints were identified based on self-reported medical history data from the NHANSE database.

## Statistical Analysis

Descriptive statistics were conducted to summarize the baseline characteristics of the study population. Continuous variables are expressed as mean ± standard deviation, while categorical variables are expressed as frequencies and percentages. The chi-square test was used to assess differences in the TyG index with categorical variables, and ANOVA was used to assess differences in means across TyG index quartiles. The sensitivity and specificity of the TyG index in predicting the endpoints of interest were evaluated using receiver operating characteristic (ROC) analysis. The negative predictive value (NPV) and positive predictive value (PPV) were calculated to evaluate the performance of the TyG index as a screening tool for endpoints of interest. Univariate and multivariate binary logistic regression analyses were conducted through multiple models to study the predictive relationship of TyG index and the endpoints of interest in this study. All statistical analyses were performed using the Statistical Package for the Social Sciences (SPSS version 25). Statistical significance was set at *P* < 0.05.

## Results

### Baseline Sociodemographic, Laboratory and History Characteristics

Twenty-eight of the thirty-two variables were significantly associated with TyG index values (*p* < 0.05) (Table 1). Most study participants were non-Hispanic white, with a mean age of 48 ± 18 years, and approximately 52.4% were female. Participants with a TyG index > 8.96 tended to have higher BMI and waist circumference (30.6 ± 6.19, 104.13 ± 62, respectively, *p* < 0.001) than those with a lower TyG index value. Direct HDL-Cholesterol levels were higher in participants with a TyG index < 8.1 (62 ± 17 mg/dl) than in those with a higher TyG index value (p < 0.001). Individuals with a TyG index > 8.96 had higher systolic/diastolic blood pressure, total cholesterol levels, LDL levels, and Apo-B levels than in those with a lower TyG index value (all p < 0.001). However, alcohol consumption was not associated with the TyG index value (p = 0.44). Being overweight and having CHF, heart attack, stroke, coronary heart disease, angina, and high blood pressure were all significantly associated with TyG index (*p* < 0.05) (Table 1).

**Table 1:** Baseline, laboratory and history characteristics of the included participants (10,937)

Variable	Categories	TYG Index ranges					*P* value
		< = 8.11	8.12—8.52	8.53—8.95	8.96 +	Total	
Age (Mean ± SD)		42 ± 18	49 ± 18	51 ± 18	51 ± 18	48 ± 18	< 0.001
Gender	Male	1085 (39.6%)	1289(47.3%)	1340(48.9%)	1491 (54.5%)	5205 (47.6%)	< 0.001
	Female	1652 (60.4%)	1435 (52.7%)	1402 (51.1%)	1243 (45.5%)	5732 (52.4%)	
Race/Ethnicity	Mexican American	316 (11.5%)	404 (14.8%)	498 (18.2%)	606 (22.2%)	1824 (16.7%)	< 0.001
	Other Hispanic	143 (5.2%)	178 (6.5%)	188 (6.9%)	180 (6.6%)	689 (6.3%)	
	Non-Hispanic White	1009 (36.9%)	1209 (44.4%)	1307 (47.7%)	1398 (51.1%)	4923 (45.0%)	
	Non-Hispanic Black	941 (34.4%)	686 (25.2%)	452 (16.5%)	268 (9.8%)	2347 (21.5%)	
	Other Race—Including Multi-Racial	328 (12.0%)	247 (9.1%)	297 (10.8%)	282 (10.3%)	1154 (10.6%)	
Education Level—Adults 20 +	Less Than 9th Grade	146 (5.3%)	233 (8.6%)	301 (11.0%)	410 (15.0%)	1090 (10.0%)	< 0.001
	9-11th Grade (Includes 12th grade with no diploma)	311 (11.4%)	349 (12.8%)	411 (15.0%)	363 (13.3%)	1434 (13.1%)	
	High School Grad/GED or Equivalent	614 (22.5%)	661 (24.3%)	662 (24.2%)	672 (24.6%)	2609 (23.9%)	
	Some College or AA degree	961 (35.2%)	870 (32.0%)	761 (27.8%)	766 (28.1%)	3358 (30.7%)	
	College Graduate or above	701 (25.6%)	610 (22.4%)	601 (22.0%)	519 (19.0%)	2431 (22.3%)	
Marital Status	Married	1320 (48.2%)	1476 (54.2%)	1563 (57.1%)	1675 (61.3%)	6034 (55.2%)	< 0.001
	Widowed	278 (10.2%)	324 (11.9%)	355 (13.0%)	322 (11.8%)	1279 (11.7%)	
	Divorced	448 (16.4%)	350 (12.9%)	305 (11.1%)	272 (10.0%)	1375 (12.6%)	
	Separated	47 (1.7%)	52 (1.9%)	70 (2.6%)	54 (2.0%)	223 (2.0%)	
	Never married	467 (17.1%)	360 (13.2%)	294 (10.7%)	237 (8.7%)	1358 (12.4%)	
	Living with partner	176 (6.4%)	161 (5.9%)	152 (5.5%)	171 (6.3%)	660 (6.0%)	
Ever had a drink of any kind of alcohol	Yes	1447 (91.2%)	1091 (90.5%)	995 (92.1%)	815 (90.3%)	4348 (91.1%)	0.446
	No	140 (8.8%)	114 (9.5%)	85 (7.9%)	88 (9.7%)	427 (8.9%)	
Usually work 35 or more hours per week	Yes	188 (38.3%)	178 (40.0%)	167 (38.9%)	183 (43.2%)	716 (40.0%)	0.463
	No	303 (61.7%)	267 (60.0%)	262 (61.1%)	241 (56.8%)	1073 (60.0%)	
Vigorous work activity	Yes	920 (38.5%)	833 (37.9%)	689 (33.2%)	644 (33.8%)	3086 (36.0%)	< 0.001
	No	1470 (61.5%)	1363 (62.1%)	1389 (66.8%)	1259 (66.2%)	5481 (64.0%)	
Smoking in the last 30 days	Every day	408 (39.2%)	474 (39.0%)	488 (37.7%)	481 (34.8%)	1851 (37.5%)	0.034
	Some days	101 (9.7%)	120 (9.9%)	102 (7.9%)	117 (8.5%)	440 (8.9%)	
	Not at all	532 (51.1%)	621 (51.1%)	704 (54.4%)	786 (56.8%)	2643 (53.6%)	
Dr told to take daily low-dose aspirin?	Yes	216 (26.3%)	287 (31.9%)	312 (37.6%)	232 (33.5%)	1047 (32.3%)	< 0.001
	No	604 (73.7%)	613 (68.1%)	517 (62.4%)	461 (66.5%)	2195 (67.7%)	
Taking prescription for hypertension (Antihypertensive drugs)	Yes	447 (81.9%)	655 (84.1%)	792 (85.2%)	843 (84.6%)	2737 (84.2%)	0.396
	No	99 (18.1%)	124 (15.9%)	138 (14.8%)	154 (15.4%)	515 (15.8%)	
Told to take prescription for cholesterol (Lowering cholesterol drugs)	Yes	268 (21.6%)	425 (33.3%)	558 (41.6%)	687 (47.5%)	1938 (36.5%)	< 0.001
	No	973 (78.4%)	850 (66.7%)	784 (58.4%)	759 (52.5%)	3366 (63.5%)	
60 s. pulse (30 s. pulse * 2):		68 ± 11	70 ± 12	70 ± 12	72 ± 13	70 ± 12	< 0.001
Systolic: BP (3rd rdg) mm Hg		118 ± 18	122 ± 19	124 ± 20	126 ± 19	123 ± 19	< 0.001
Diastolic: BP (3rd rdg) mm Hg		70 ± 12	72 ± 13	72 ± 13	73 ± 14	72 ± 13	< 0.001
Systolic: BP (4th rdg) mm Hg		124 ± 22	125 ± 21	127 ± 21	129 ± 18	126 ± 21	0.021
Diastolic: BP (4th rdg) mm Hg		72 ± 15	71 ± 15	71 ± 15	71 ± 19	71 ± 16	0.747
Body Mass Index (kg/m**2)		26.42 ± 6.42	28.4 ± 7.04	29.36 ± 6.88	30.60 ± 6.19	28.69 ± 6.81	< 0.001
Waist Circumference (cm)		90.02 ± 15.13	96.65 ± 15.59	99.99 ± 14.85	104.13 ± 13.63	97.71 ± 15.69	< 0.001
Direct HDL-Cholesterol (mg/dL)		62 ± 17	58 ± 16	53 ± 15	46 ± 13	54 ± 16	< 0.001
Total cholesterol (mg/dL)		174 ± 35	190 ± 37	200 ± 38	217 ± 48	195 ± 43	< 0.001
LDL-cholesterol (mg/dL)		100 ± 30	115 ± 33	123 ± 35	124 ± 40	115 ± 36	< 0.001
Apolipoprotein (B) (mg/dL)		81 ± 21	94 ± 21	106 ± 24	119 ± 29	102 ± 28	< 0.001
Albumin, urine (mg/L) SI		27.18 ± 221.80	33.43 ± 282.47	27.40 ± 114.95	44.61 ± 333.21	33.16 ± 251.65	0.036
Creatinine, urine (mg/dL)		140 ± 87	135 ± 84	137 ± 84	133 ± 75	136 ± 83	0.020
Doctor ever said you were overweight	Yes	597 (21.8%)	786 (28.9%)	912 (33.3%)	1071 (39.2%)	3366 (30.8%)	< 0.001
	No	2137 (78.2%)	1936 (71.1%)	1827 (66.7%)	1661 (60.8%)	7561 (69.2%)	
Ever told you had high blood pressure	Yes	548 (20.1%)	779 (28.8%)	930 (34.1%)	997 (36.7%)	3254 (29.9%)	< 0.001
	No	2177 (79.9%)	1926 (71.2%)	1798 (65.9%)	1721 (63.3%)	7622 (70.1%)	
Ever told had congestive heart failure	Yes	42 (1.5%)	53 (1.9%)	63 (2.3%)	79 (2.9%)	237 (2.2%)	0.005
	No	2688 (98.5%)	2669 (98.1%)	2672 (97.7%)	2641 (97.1%)	10,670 (97.8%)	
Ever told you had coronary heart disease	Yes	34 (1.2%%)	80 (2.9%)	119 (4.4%)	127 (4.7%)	360 (3.3%)	< 0.001
	No	2695 (98.8%)	2639 (97.1%)	2609 (95.6%)	2598 (95.3%)	10,541 (96.7%)	
Ever told you had angina/angina pectoris	Yes	30 (1.1%)	37 (1.4%)	91 (3.3%)	99 (3.6%)	257 (2.4%)	< 0.001
	No	2699 (98.9%)	2678 (98.6%)	2643 (96.7%)	2627 (96.4%)	10,647 (97.6%)	
Ever told you had heart attack	Yes	38 (1.4%)	91 (3.3%)	118 (4.3%)	138 (5.1%)	385 (3.5%)	< 0.001
	No	2695 (98.6%)	2633 (96.7%)	2621 (95.7%)	2591 (94.9%)	10,540 (96.5%)	
Ever told you had a stroke	Yes	61(2.2%)	93 (3.4%)	104 (3.8%)	101 (3.7%)	359 (3.3%)	0.004
	No	2671 (21.8%)	2630 (28.9%)	2638 (96.2%)	2628 (96.3%)	10,567 (96.7%)	

## Regression Analysis of the Association between Coronary Heart Disease & TyG Index

When adjusting for age, sex, race, and BMI, we found that an increase in the TyG index value was associated with an increased likelihood of coronary heart disease. Participants with a TyG index > 8.96 were 2.24 times more likely to have coronary heart disease than those with a TyG index < 8.11 (95% CI = 1.48–3.41, *p* < 0.001). Additionally, after adjusting for stroke, overweight, heart attack, CHF, and hypertension, higher TyG index value was still positively associated with having coronary heart disease. For instance, individuals with a TyG index between 8.53–8.95 had 2.54 times higher odds for coronary heart disease than those with a TyG index < 8.11 (95% CI = 1.63–3.94, *p* < 0.001). When adjusting for cholesterol, LDL, Apo-B, creatinine, and urine albumin, participants with a TyG index between 8.53–8.95 were 6.45 times more likely to have coronary heart disease than those with a TyG index < 8.11 (AOR = 6.45, 95% CI = 1.8–23.09, *p* = 0.004) (Table 2).

**Table 2:** Regression analysis of the association between coronary heart disease & TyG index

Variable	Categories	Ever been told you had coronary heart disease Yes (*n*=360)\No (*n*=10541)							
		Model 1				Model 2			
		*P*-Value	AOR	95% C.I. for AOR		*P*-Value	COR	95% C.I. for COR	
				Lower	Upper			Lower	Upper
TyG Index	<= 8.11	Ref				Ref			
	8.12–8.52	0.033	1.597	1.039	2.454	0.000	2.403	1.603	3.602
	8.53–8.95	0.000	2.119	1.400	3.209	0.000	3.615	2.460	5.313
	8.96+	0.000	2.249	1.480	3.417	0.000	3.875	2.644	5.679
		Adjusted for Age, sex, race and BMI				Unadjusted Model			

Variable	Categories	Ever told you had coronary heart disease Yes (*n*=360)\No (*n*=10541)							
		Model 3				Model 4			
		*P* Value	AOR	95% C.I. for AOR		*P* Value	AOR	95% C.I. for COR	
				Lower	Upper			Lower	Upper
TyG Index	<= 8.11	Ref				Ref			
	8.12–8.52	0.540	2.012	0.215	18.843	0.045	2.080	1.018	4.248
	8.53–8.95	0.333	2.933	0.333	25.847	0.127	1.730	0.855	3.499
	8.96+	0.172	4.521	0.517	39.501	0.498	1.298	0.611	2.758
		Adjusted for time of working, Vigorous activity, Alcohol and smoking status				Adjusted for telling the patients to take Aspirin, Antihypertensive drugs and lowering cholesterol drugs			

Variable	Categories	Ever told you had coronary heart disease Yes (*n*=360)\No (*n*=10541)							
		Model 5				Model 6			
		*P* Value	AOR	95% C.I. for AOR		*P* Value	AOR	95% C.I. for COR	
				Lower	Upper			Lower	Upper
TyG Index	<= 8.11	Ref				Ref			
	8.12–8.52	0.022	1.713	1.080	2.719	0.021	4.484	1.248	16.106
	8.53–8.95	0.000	2.544	1.639	3.948	0.004	6.458	1.806	23.090
	8.96+	0.000	2.257	1.450	3.515	0.017	5.589	1.352	23.105
		Adjusted for Stroke, Overweight, heart attack, congestive heart failure and Hypertension				Adjusted for Cholesterol, LDL, Apolipoprotein B, urine creatinine and urine albumin			

## Regression Analysis of the Association between Stroke & TyG Index

After adjusting our findings for cholesterol, LDL, Apo-B, creatinine, and urine albumin, participants with a TyG index > 8.96 were 4.42 times more likely to suffer from a stroke than those with a TyG index < 8.11 (AOR = 4.42, 95% CI = 1.28–15.24, *p* = 0.01), however unadjusted regression model reported that participants with a TyG index > 8.96 were 1.68 times more likely to suffer from a stroke than those with a TyG index < 8.11 (AOR = 1.68, 95% CI = 1.21–2.32, *p* = 0.002) (Table 3).

**Table 3:** Regression analysis of the association between stroke & TyG index

Variable	Categories	Ever told you had a stroke Yes (*n*=359)\No (*n*=10567)							
		Model 1				Model 2			
		*P* Value	AOR	95% C.I. for AOR		*P* Value	COR	95% C.I. for COR	
				Lower	Upper			Lower	Upper
TyG Index	<= 8.11	Ref				Ref			
	8.12–8.52	0.469	1.136	0.804	1.604	0.009	1.548	1.116	2.148
	8.53–8.95	0.613	1.094	0.772	1.551	0.001	1.726	1.253	2.379
	8.96+	0.224	1.246	0.874	1.777	0.002	1.683	1.219	2.323
		Adjusted for Age, sex, race and BMI				Unadjusted Model			

Variable	Categories	Ever told you had a stroke Yes (*n*=359)\No (*n*=10567)							
		Model 3				Model 4			
		*P* Value	AOR	95% C.I. for AOR		*P* Value	AOR	95% C.I. for COR	
				Lower	Upper			Lower	Upper
TyG Index	<= 8.11	Ref				Ref			
	8.12–8.52	0.741	0.732	0.115	4.652	0.210	1.438	0.815	2.535
	8.53–8.95	0.370	0.386	0.048	3.092	0.749	1.098	0.621	1.942
	8.96+	0.763	0.747	0.111	5.010	0.666	1.139	0.630	2.059
		Adjusted for time of working, Vigorous activity, Alcohol and smoking status				Adjusted for telling the patients to take Aspirin, Antihypertensive drugs and lowering cholesterol drugs			

Variable	Categories	Ever told you had a stroke Yes (*n*=359)\No (*n*=10567)							
		Model 5				Model 6			
		*P* Value	AOR	95% C.I. for AOR		*P* Value	AOR	95% C.I. for COR	
				Lower	Upper			Lower	Upper
TyG Index	<= 8.11	Ref				Ref			
	8.12–8.52	0.123	1.308	0.930	1.840	0.748	1.212	0.375	3.921
	8.53–8.95	0.140	1.289	0.920	1.807	0.136	2.325	0.767	7.049
	8.96+	0.449	1.142	0.810	1.611	0.018	4.429	1.287	15.244
		Adjusted for Stroke, Overweight, heart attack, congestive heart failure and Hypertension				Adjusted for Cholesterol, LDL, Apolipoprotein B, urine creatinine and urine albumin			

## Regression Analysis of the Association between Heart Attack & TyG Index

When studying the association between heart attack and the TyG index, after adjusting for age, sex, race, and BMI, we found that as the TyG index value increased, the likelihood of having a heart attack also increased. Furthermore, adjusting our results for taking aspirin, antihypertensive, and cholesterol-lowering drugs showed a statistically significant association between having a heart attack and a TyG index between 8.53–8.95 (AOR = 2.2, 95% CI = 1.08–4.47, p = 0.029). We found that a higher TyG index was significantly associated with higher odds of having a heart attack after adjusting for stroke, overweight, heart attack, CHF, and hypertension. Individuals with a TyG index > 8.96 were 2.45 times more likely to have a heart attack than individuals with an index < 8.11 (AOR = 2.45, 95% CI = 1.59–3.77, P < 0.001) (Table 4).

**Table 4:** Regression analysis of the association between heart attack & TyG index

Variable	Categories	Ever told you had heart attack Yes (*n*=385)\No (*n*=10540)							
		Model 1				Model 2			
		*P* Value	AOR	95% C.I. for AOR		*P* Value	COR	95% C.I. for COR	
				Lower	Upper			Lower	Upper
TyG Index	<= 8.11	Ref				Ref			
	8.12–8.52	0.009	1.716	1.146	2.571	0.000	2.451	1.672	3.593
	8.53–8.95	0.000	2.048	1.378	3.044	0.000	3.193	2.207	4.620
	8.96+	0.000	2.451	1.648	3.645	0.000	3.777	2.627	5.431
		Adjusted for Age, sex, race and BMI				Unadjusted Model			

Variable	Categories	Ever told you had heart attack Yes(n=385) \ No(n=10540)							
		Model 3				Model 4			
		*P* Value	AOR	95% C.I. for AOR		*P* Value	AOR	95% C.I. for COR	
				Lower	Upper			Lower	Upper
TyG Index	<= 8.11	Ref				Ref			
	8.12–8.52	0.873	1.152	0.203	6.552	0.090	1.915	0.905	4.052
	8.53–8.95	0.837	0.826	0.134	5.104	0.029	2.203	1.084	4.475
	8.96+	0.709	0.695	0.103	4.698	0.097	1.888	0.891	4.002
		Adjusted for time of working, Vigorous activity, Alcohol and smoking status				Adjusted for telling the patients to take Aspirin, Antihypertensive drugs and lowering cholesterol drugs			

Variable	Categories	Ever told you had heart attack Yes (*n*=385)\No (*n*=10540)							
		Model 5				Model 6			
		*P* Value	AOR	95% C.I. for AOR		*P* Value	AOR	95% C.I. for COR	
				Lower	Upper			Lower	Upper
TyG Index	<= 8.11	Ref				Ref			
	8.12 - 8.52	0.002	2.030	1.301	3.168	0.105	2.410	0.833	6.972
	8.53 - 8.95	0.000	2.207	1.430	3.405	0.098	2.475	0.847	7.237
	8.96+	0.000	2.451	1.592	3.775	0.046	3.339	1.020	10.932
		Adjusted for Stroke, Overweight, heart attack, congestive heart failure and Hypertension				Adjusted for Cholesterol, LDL, Apolipoprotein B, urine creatinine and urine albumin			

When adjusting our regression analysis for age, sex, race, and BMI, we observed that higher TyG index values were associated with higher odds of hypertension. Participants with a TyG index between 8.53–8.95 had higher odds for hypertension than those with a TyG index < 8.11 (AOR = 1.23, 95% CI = 1.07–1.42, p = 0.003). The same trend of association was also reported after adjusting for stroke, overweight, heart attack, CHF, and hypertension. Participants with a TyG index > 8.96 were 1.89 times more likely to have hypertension than those with a TyG index < 8.11 (AOR = 1.89, 95% CI = 1.66–2.15, P < 0.001). Similarly, an increased TyG index value correlated with an increased likelihood of hypertension. For instance, participants with a TyG index had a higher likelihood of having hypertension than those with a TyG index < 8.11 (AOR = 3.93, 95% CI = 2.55–6.08, P < 0.001) (Table 5).

**Table 5:** Regression analysis of the association between hypertension & TyG index

Variable	Categories	Ever told you had high blood pressure Yes (*n*=3254)\No (*n*=7622)							
		Model 1				Model 2			
		*P* Value	AOR	95% C.I. for AOR		*P* Value	COR	95% C.I. for COR	
				Lower	Upper			Lower	Upper
TyG Index	<= 8.11	Ref				Ref			
	8.12 - 8.52	0.003	1.239	1.075	1.429	0.000	1.607	1.418	1.821
	8.53 - 8.95	0.000	1.539	1.334	1.775	0.000	2.055	1.818	2.323
	8.96+	0.000	1.754	1.517	2.029	0.000	2.301	2.037	2.600
		Adjusted for Age, sex, race and BMI				Unadjusted Model			

Variable	Categories	Ever told you had high blood pressure Yes (*n*=3254)\No (*n*=7622)							
		Model 3				Model 4			
		*P* Value	AOR	95% C.I. for AOR		*P* Value	AOR	95% C.I. for COR	
				Lower	Upper			Lower	Upper
TyG Index	<= 8.11	Ref				Ref			
	8.12–8.52	0.240	1.764	0.684	4.550	0.242	0.876	0.703	1.093
	8.53–8.95	0.276	1.720	0.648	4.568	0.228	1.148	0.917	1.438
	8.96+	0.093	2.310	0.870	6.134	0.830	1.026	0.812	1.296
		Adjusted for time of working, Vigorous activity, Alcohol and smoking status				Adjusted for telling the patients to take Aspirin and lowering cholesterol drugs			

Variable	Categories	Ever told you had high blood pressure Yes (*n*=3254)\No (*n*=7622)							
		Model 5				Model 6			
		*P* Value	AOR	95% C.I. for AOR		*P* Value	AOR	95% C.I. for COR	
				Lower	Upper			Lower	Upper
TyG Index	<= 8.11	Ref				Ref			
	8.12–8.52	0.000	1.470	1.290	1.675	0.000	2.586	1.792	3.732
	8.53–8.95	0.000	1.798	1.581	2.045	0.000	3.149	2.162	4.587
	8.96+	0.000	1.894	1.666	2.154	0.000	3.937	2.550	6.080
		Adjusted for Stroke, Overweight, heart attack, congestive heart failure and Hypertension				Adjusted for Cholesterol, LDL, Apolipoprotein B, urine creatinine and urine albumin			

### The Predictive Value of the TyG for Cardiovascular Diseases

**Fig. 1:**
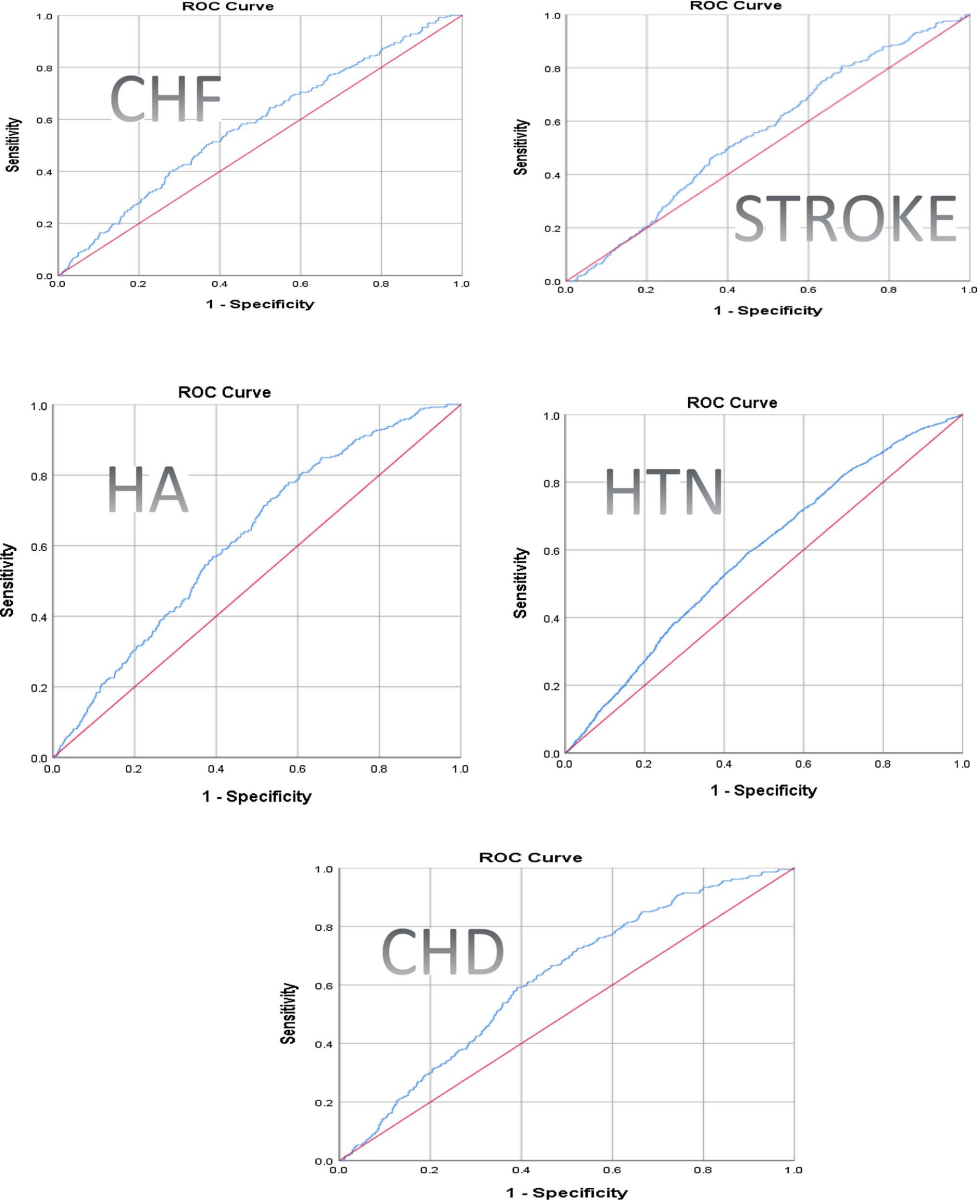
ROC curve of the association between cardiovascular diseases incidence and the value of TYG index

The TyG index was a poor indicator for all studied variables (CHF, stroke, heart attack, high blood pressure, and coronary heart disease) (*p* < 0.05). The TyG index was a poor indicator for predicting CHF (AUC = 54.75, 95% CI = 0.54–0.61) with a cut-off value of 8.58, 58.23% sensitivity, 54.68% specificity, 2.8% PPV, and 98.31% NPV. The TyG index was a better indicator for predicting a heart attack (AUC = 55.67%, 95% CI = 0.59–0.64) than for predicting stroke (AUC = 52.32%, 95% CI = 0.52–0.58), with cut-off values of 8.59 and 8.54, respectively. The TyG index was a poor indicator for predicting high blood pressure (AUC = 55.59%, 95% CI = 0.57–0.59) with a cut-off value of 8.52, 58.94% sensitivity, 54.16% specificity, 35.42% PPV, and an NPP of 75.57%. The use of the TyG index to predict coronary heart disease status was insufficient, as the AUC was 50.31% (95% CI = 0.59–0.64) with a cut-off value of 8.5031, 67.17% sensitivity, 49.66% specificity, 4.47% PPV, and an NPV of 97.93% (Table 6) (Fig. 1) C.

**Table 6:** Assessment of the value of the TYG index in prediction of the studied diseases

Diagnostic method	Result	Suggestive of n (%)	Not Suggestive of n (%)	Total n (%)	*P* value	Sensitivity Specificity	PPV NPV	AUC	Asymptotic 95% Confidence Interval	
									Lower Bound	Upper Bound
Congestive heart failure										
TYG	High	138 (2.8)	4836 (97.2)	4974 (45.6)	< 0.001	58.23% 54.68%	2.809% 98.31%	54.75%	0.542	0.614
	Low	99 (1.7)	5834 (98.3)	5933 (54.4)						
	Total	237 (2.2)	10,670 (97.8)	10,907 (100)						
Stroke										
TYG	High	202 (3.8)	5052 (96.2)	5254 (48.1)	0.002	56.27% 62.19%	3.861% 97.22%	52.32%	0.529	0.584
	Low	157(2.8)	5515 (97.2)	5672 (51.9)						
	Total	359(3.3)	10,567 (96.7)	10,926(100)						
Heart attack										
TYG	High	236 (4.7)	4694 (95.2)	4930 (45.1)	< 0.001	61.3% 55.46%	4.755% 97.53%	55.67%	0.595	0.646
	Low	149 (2.5)	5846 (97.5)	5995 (54.9)						
	Total	385 (3.5)	10,540 (96.5)	10,925 (100)						
High blood pressure										
TYG	High	1918 (35.4)	3494 (64.6)	5412 (49.8)	< 0.001	58.94% 54.16%	35.42% 75.57%	55.59%	0.574	0.597
	Low	1336 (24.5)	4128 (75.5)	5464 (50.2)						
	Total	3254 (29.9)	7622 (70.1)	10,876 (100)						
Coronary heart disease										
TYG	High	249 (4.5)	5306 (95.5)	5555 (51)	< 0.001	69.17% 49.66%	4.479% 97.93%	50.31%	0.592	0.646
	Low	111 (2.1)	5235 (97.9)	5346 (49)						
	Total	360 (3.3)	10,541 (96.7)	10,901 (100)

## Discussion

The TyG index serves as a useful indicator of insulin resistance, determined by the natural logarithm of fasting triglycerides multiplied by fasting plasma glucose, rendering it appropriate for use in primary care settings equipped with laboratory services. [11] Insulin resistance (IR) denotes a condition where the efficacy and regulatory mechanisms of insulin-mediated glucose metabolism are disrupted across multiple tissues, reflecting a significant physiological anomaly. [12] IR contributes to the onset of several metabolic disorders, such as hyperglycemia, hypertension, and dyslipidemia, which are closely linked to diabetes, atherosclerosis, and cardiovascular diseases. [13] Studies have indicated a correlation between the TyG index and cardiovascular diseases (CVDs). Specifically, individuals without diabetes mellitus, elevated TyG index values were associated with a higher probability of cardiovascular disorders. [14].

The present analysis employs NHANES data obtained from 2001 to 2020, with the aim of evaluating the TyG index's precision in predicting cardiovascular diseases among patients without diabetes mellitus.

Our analysis revealed that a TyG index exceeding 8.96 significantly increased the likelihood of coronary heart disease compared to a TyG index below 8.11. In model 1, the adjusted odds ratio (AOR) was 2.24, and model 5 yielded an AOR of 2.54. Nonetheless, the TyG index's predictive value for coronary heart disease was minimal, as indicated by an AUC of 50.31% (95% CI 0.59–0.64), a cutoff value of 8.5031, 67.17% sensitivity and 49.66% specificity.

A study indicated that the TyG index was correlated with a heightened probability of major adverse cardiovascular events (MACEs), with this probability increasing as the TyG index rose (TyG between 8.48 and 8.85: OR: 1.001; TyG between 8.85 and 9.18: OR: 1.04; TyG above 9.18: OR: 1.04) [15]. Furthermore, individuals with the highest TyG index exhibited a 1.47 times greater risk of MACEs over a one-year follow-up period [15]. In a separate 16-year longitudinal study, participants with elevated TyG levels demonstrated a 36% higher risk of developing cardiovascular disease compared to those with lower TyG levels (hazard ratio, 1.36) [16]. Another investigation involving 5014 subjects found that individuals with a higher TyG index had a 2.32-fold increased risk of cardiovascular disease [17]. Additionally, a systematic review concluded that patients with elevated TyG indices had a higher risk of coronary artery disease (CAD) (OR: 1.94) in comparison to those with lower TyG indices [18]. Patients with higher TyG levels were also more prone to having stenotic coronary arteries (OR: 3.49), more advanced plaques (OR: 1.67), and a greater number of affected vessels (OR: 2.33) [18]. Moreover, among patients with chronic coronary syndrome (CCS) or stable CAD, those with higher TyG index levels exhibited a trend towards a higher incidence of MACEs (HR: 1.24) [18].

A study examining 6463 patients revealed that the TyG-index had an AUC of 0.633 (95% CI: 0.645–0.681) for predicting CVD incidence and 0.669 (95% CI: 0.651–0.688) for CHD incidence. The optimal cutoff value for the TyG-index in detecting CVD was 9.03, with a sensitivity of 59.23% and specificity of 63.15%; for CHD, the same cutoff value yielded a sensitivity of 59.96% and specificity of 62.84% [19]. Another research study demonstrated that the AUC of the TyG index for predicting MACCEs was 0.604 (95% CI: 0.578–0.630; *p* < 0.001), with a cutoff value of 9.30, sensitivity of 0.552, and specificity of 0.613 [20].

Insulin resistance (IR) is associated with the onset of diabetes and cardiovascular diseases (CVDs). Even healthy adults may exhibit IR, which is a pivotal factor in the pathogenesis of atherosclerosis and coronary artery disease (CAD). IR can lead to abnormal metabolism and, when combined with glucose intolerance, increases the risk of CAD. Dysregulated insulin signaling can impair nitric oxide production, contributing to vascular rigidity. Factors such as nonenzymatic glycosylation of lipids, hypertriglyceridemia, and hyperglycemia play roles in the development of atherosclerosis. IR also augments sympathetic nervous system activity, renal sodium retention, and blood pressure while inducing metabolic effects that synergistically impair vascular and renal systems. Inadequate insulin signaling can activate systems that compromise cardiac function, and IR is linked to decreased cardiac autonomic function in non-diabetic patients [18].

Our study findings indicated that participants with a TyG index > 8.96 had a greater likelihood of stroke than those with a TyG index < 8.11 (model 6, adjusted for cholesterol, LDL, Apo-B, creatinine, and urine albumin: adjusted odds ratio = 4.42). The TyG index was identified as an insufficient indicator for predicting stroke (AUC = 52.32%, 95% CI = 0.52–0.58), with a threshold of 8.54.

A study involving 10,132 participants revealed that individuals in the lowest quartile of the baseline TyG index (8.0 ± 0.2) had a heightened risk of incident stroke compared to those in the highest quartile (9.4 ± 0.4) (HR: 1.254) [21]. Another investigation indicated an escalating risk of stroke with increasing TyG levels, showing hazard ratios of 1.04, 1.43, and 1.45 in quartiles 2 (TyG: 8.5– < 8.8), 3 (TyG: 8.8– < 9.2), and 4 (TyG: ≥ 9.2), respectively, compared to quartile 1 (TyG: < 8.5) [22]. A study involving 17,708 middle-aged and elderly participants found that those with the highest TyG index had a 1.76 times higher incidence of stroke compared to those with the lowest TyG index [23]. Similarly, a study of 2,288 participants demonstrated that individuals with the highest TyG index had a greater risk of recurrent stroke compared to those with the lowest TyG index (HR = 1.63) [24]. A systematic review revealed a positive association between a higher TyG index and an increased risk of ischemic stroke (IS) in the general population (OR 1.37). Furthermore, IS patients with a higher TyG index were associated with elevated risks of stroke recurrence (OR: 1.50) and mortality (OR 1.40) compared to those with a lower TyG index [24]. A study involving 955 patients reported that the area under the curve for predicting stroke recurrence using the TyG index was 0.719 (95% confidence interval, 0.66–0.77) [25].

Insulin resistance (IR) contributes significantly to the development of ischemic stroke (IS) through multiple mechanisms. IR disrupts insulin signaling, promotes chronic systemic inflammation, reduces insulin sensitivity, and increases foam cell formation, thereby accelerating atherosclerosis and advanced plaque formation. Additionally, IR alters the metabolism of insulin-like growth factor-1 (IGF-1), insulin-like growth factor-2 (IGF-2), cyclic guanosine monophosphate (cGMP), and nitric oxide (NO), influencing platelet adhesion, activation, and aggregation. These processes contribute to vascular occlusion and the pathogenesis of IS. Moreover, IR may impact cerebrovascular reserve (CVR) through myogenic, chemical, neuronal, and metabolic mechanisms, leading to impaired cerebral perfusion hemodynamics during acute IS [26].

Our analysis indicated that participants with a TyG index > 8.96 were at higher risk for heart attack compared to those with a TyG index < 8.11 (model 1: AOR = 2.45; model 5: AOR = 2.45; model 6: AOR = 3.33). Furthermore, our findings revealed that individuals with a TyG index ranging from 8.53 to 8.95 had an increased likelihood of developing coronary heart disease compared to those with a TyG index < 8.11 (model 4: AOR = 2.2). The TyG index was identified as a poor predictor of heart attack (AUC = 55.67%, 95% CI = 0.59–0.64) with a cutoff value of 8.59.

A study involving 6,695 participants demonstrated that an elevated TyG index was significantly linked to an increased risk of myocardial infarction (MI) among adults in the United States, with an odds ratio (OR) of 1.69 [27]. Additionally, another investigation indicated that the risk of MI rose across quartiles of baseline and updated mean TyG index, with hazard ratios (HRs) in quartile 4 (9.46; 95% CI: 9.23–9.82) versus quartile 1 (7.91; 95% CI: 7.70–8.06) of 2.08 and 1.58, respectively [28]. Moreover, a study identified a significant association between the TyG index and the severity of coronary stenosis, with the TyG index acting as an independent risk factor for its severity (OR 2.003) [29]. In addition, a study involving 3,181 patients revealed that in patients with acute myocardial infarction (AMI), a higher TyG index was positively correlated with all-cause death (HR: 1.51), cardiac death (HR: 1.68), cardiac rehospitalization (HR: 1.25), and composite major adverse cardiovascular events (MACEs) (HR: 1.19) [30]. Another study conducted among the US population found a significant association between a higher TyG index and increased MI risk (OR: 1.69) [27]. Furthermore, a meta-analysis indicated that a higher TyG index was associated with a higher incidence of MI (HR = 1.36) compared to the lower TyG index category [31]. However, a study demonstrated that the TyG index had a greater predictive capacity for cardiovascular diseases (CVDs) than the homeostasis model assessment of insulin resistance (HOMA-IR) (area under the curve, 0.578 for TyG and 0.543 for HOMA-IR) [16].

Insulin resistance is characterized by a mild systemic inflammatory state, leading to endothelial dysfunction. In liver and adipose tissues, insulin resistance drives the development of atherosclerotic dyslipidemia and triggers a low-grade inflammatory response, increasing the release of inflammatory markers. It also impacts blood pressure, endothelial cells, and macrophages. Concurrently, insulin resistance contributes to atherosclerosis and plaque progression through multiple pathways, including alterations in traditional cardiovascular risk factors and the suppression of insulin signaling pathways. Furthermore, the TyG index, which closely correlates with several cardiovascular disease risk factors, including coronary artery calcification (CAC), provides evidence of coronary artery disease (CAD) development.

According to our analysis, individuals with a TyG index greater than 8.96 exhibited a higher likelihood of hypertension compared to those with a TyG index lower than 8.11. In model 1, the adjusted odds ratio (AOR) was 1.75. In model 5, the AOR was 1.89. The TyG index was found to be an inadequate predictor for elevated blood pressure (AUC = 55.59%, 95% CI = 0.57–0.59) with a threshold value of 8.52, sensitivity of 58.94%.

A study demonstrated that individuals with a TyG index greater than 8.41 exhibited higher odds of hypertension (OR = 1.17) compared to those with a TyG index ≤ 8.41 [32]. In another study, participants with a higher TyG index (9.3: 9.1–9.6) were found to have an increased risk (HR = 1.56) of hypertension compared to those with a lower TyG index (8.2: 8.0–8.3) [33]. An analysis involving 16,793 participants revealed that a higher TyG index was linked to increased odds of isolated systolic hypertension (IDH) (OR = 2.94) and systolic-diastolic hypertension (SDH) (OR = 1.82) [34]. A meta-analysis indicated that an elevated TyG index was associated with a higher risk of developing hypertension, with a HR of 1.36. Furthermore, this meta-analysis showed that every one-unit increase in the TyG index was associated with a 1.5-fold increased risk of developing hypertension. Moreover, the study reported that the predictive ability of the TyG index for hypertension was inadequate across all participants (AUC, 0.583 [95% CI, 0.56–0.59]) [35].

Insulin resistance and elevated blood pressure are interconnected through multiple mechanisms. Initially, during the early stages of diabetes, compensatory hyperinsulinemia stimulates sympathetic nerves and boosts renin secretion, resulting in elevated blood pressure. This hypertension further intensifies the sympathetic response, establishing a cycle of increased blood pressure. Hyperinsulinemia enhances sodium reabsorption and raises intrarenal pressure, which diminishes urine outflow and expands body fluid volume, thereby fostering hypertension. In later stages of diabetes, persistent insulin resistance, along with hyperglycemia and oxidative stress, impairs vasodilator production and response, contributing to sustained hypertension. Hyperinsulinemia and aldosterone elevate sodium channel activity in vascular cells, while cardiovascular inflammation hinders insulin signaling and diminishes nitric oxide production, promoting arterial stiffness and hypertension [36].

Our American dataset analysis could be depended on to develop more accurate screening tools or even to guide lifestyle interventions that might prevent the onset of debilitating conditions, especially cardiovascular disorders. Overall, this research represents an encouraging advancement in our understanding of cardiovascular health beyond conventional markers such as blood pressure or cholesterol levels.

This study has some limitations, including the fact that it is cross-sectional, which means that a causal relationship cannot be established. Additionally, the data collected were based on self-reports, which may be subject to recall bias, especially in the older population. Additionally, the COVID-19 pandemic led to the suspension of NHANES 2019–2020 field activities in March 2020, after data had been collected from 18 of the 30 survey sites in the 2019–2020 sample. Data from the previous cycle (2017–2018) were combined with the collected data, which were not nationally representative, to create a pre-pandemic data file covering the period from 2017 to March 2020. This pre-pandemic data set underwent a unique weighting procedure. Neither the 2017–2018 data alone nor the 2019 to March 2020 data alone will yield nationally representative results using these sample weights, nor are they suitable for independent analysis of the 2019–2020 data. Also, our analysis process didn't exclude some endocrine disorders that could interfere with the value of TyG, such as hypothyroidism and Cushing's disease, and we didn't eliminate the patients who were on anti-lipid treatment as almost patients with any cardiovascular disease take preventive anti-lipid drugs. However, this study is representative of the American population, and although it cannot be generalized to all populations and age groups, it remains valuable. Future studies should be conducted in different populations, ethnicities, and age groups to further understand this topic.

## Conclusions

This study emphasizes the significance of TyG index as an additional tool for predicting CVD risk in patients without diabetes mellitus. The TyG index demonstrated a strong correlation with cardiovascular risk factors in individuals without diabetes. However, it was a poor predictor for nearly all the cardiovascular diseases studied. This serves as a starting point to broaden our understanding of cardiovascular health beyond conventional risk markers. However, further investigation is crucial to comprehend the mechanism, authentication, and refinement of the clinical utility of the TyG index, ultimately contributing to more effective prevention and management strategies for cardiovascular diseases.

## Data Availability

The datasets generated and/or analysed during the current study are available in the National Health and Nutrition Examination Survey repository, [https://wwwn.cdc.gov/nchs/nhanes/continuousnhanes/default.aspx].
